# Implementing evidence-based medicine in general practice: a focus group based study

**DOI:** 10.1186/1471-2296-6-37

**Published:** 2005-09-09

**Authors:** Karin Hannes, Marcus Leys, Etienne Vermeire, Bert Aertgeerts, Frank Buntinx, Anne-Marie Depoorter

**Affiliations:** 1 Belgian Centre for Evidence-Based Medicine-Belgian Branch of the Cochrane Collaboration, Kapucijnenvoer 33 blok J, 3000 Leuven, Belgium; 2 Department of Medical Sociology Vrije Universiteit Brussel, Laerbeeklaan 103, 1090 Jette, Belgium; 3 Department of General Practice, Universiteit Antwerpen, Universiteitsplein 1, 2610 Antwerpen, Belgium; 4 Academic Centre for General Practice, Katholieke Universiteit Leuven, Kapucijnenvoer 33 blok J, 3000 Leuven, Belgium; 5 Department of Public Health, Vrije Universiteit Brussel, Laerbeeklaan 103, 1090 Jette, Belgium

## Abstract

**Background:**

Over the past years concerns are rising about the use of Evidence-Based Medicine (EBM) in health care. The calls for an increase in the practice of EBM, seem to be obstructed by many barriers preventing the implementation of evidence-based thinking and acting in general practice. This study aims to explore the barriers of Flemish GPs (General Practitioners) to the implementation of EBM in routine clinical work and to identify possible strategies for integrating EBM in daily work.

**Methods:**

We used a qualitative research strategy to gather and analyse data. We organised focus groups between September 2002 and April 2003. The focus group data were analysed using a combined strategy of 'between-case' analysis and 'grounded theory approach'. Thirty-one general practitioners participated in four focus groups. Purposeful sampling was used to recruit participants.

**Results:**

A basic classification model documents the influencing factors and actors on a micro-, meso- as well as macro-level. Patients, colleagues, competences, logistics and time were identified on the micro-level (the GPs' individual practice), commercial and consumer organisations on the meso-level (institutions, organisations) and health care policy, media and specific characteristics of evidence on the macro-level (policy level and international scientific community). Existing barriers and possible strategies to overcome these barriers were described.

**Conclusion:**

In order to implement EBM in routine general practice, an integrated approach on different levels needs to be developed.

## Background

Over the past years concerns are rising about the use of Evidence-Based Medicine (EBM) in health care. The calls for an increase in the practice of EBM, seem to be obstructed by many barriers preventing the implementation of evidence-based thinking and acting in general practice. There is a growing need to develop strategies that are able to address the barriers towards evidence-based thinking and acting. We searched Medline, the Cochrane Library, ACP-Journal Club, Dare, Sociological abstracts, Psychinfo and the Campbell Library to reveal studies addressing barriers towards the implementation of EBM (table 1) as well as relevant literature proposing feasible interventions for an evidence-based approach in general practice (table 2) up till May 2003 [1-22]. We used the MeSH-terms Evidence-Based Medicine, Family Physician, Attitude of Health Personal and combined them with free text words like implement$, utilization, use, barrier$, obstacle$, intervent$ and strateg$. An additional hand search was conducted for the period 2000-May 2003 in the BMJ, JAMA, Lancet, New England Journal of Medicine, Annals of Internal Medicine, British Journal of General Practice, Journal of Evidence-Based Medicine and the European Journal of Family Practice. Numerous studies have suggested that a lack of time, easy accessible EBM-resources and competences (knowledge, skills, attitudes) affect the implementation of EBM, as well as the gaps in existing scientific knowledge. Also colleagues, patients and commercial organisations have an influence on the ability to think and act Evidence-Based. Existing interventions aiming to improve the implementation of EBM are limited to logistic or educational support. The review demonstrates a lack of well-controlled empirical studies identifying clear suggestions on how to bridge barriers and improve the implementation of EBM. As a consequence it becomes very difficult to make potential actors at different levels in the health care system feel responsible for developing strategies to optimise the implementation of EBM. The exploration of barriers Flemish GPs (General Practitioners) experience when implementing EBM in daily clinical practice can lead to suggestions about an approach to break down existing barriers. This study proposes a framework, based on experiences of GPs, considering the complex networks of 'actors' and 'factors' affecting the implementation of EBM.

**Table 1:** Studies addressing barriers towards EBM in general practice

**Study Year**	**Olantunbosun et al, 1998 [1]**	**Mc Coll et al 1998 [2]**	**McAlister et al 1999 [3]**	**Mayer et al 1999 [4]**	**Tomlin et al 1999 [5]**
**Population**	Randomised sample of GPs and Gynaecologists in Canada	Randomised sample of GPs in Wessex, United Kingdom	Gps, members of the 'Canadian Society of Internal Medicine, Canada	Purposeful sample of GPs of educational programs, courses, supervisors of the 'Adelaide Royal Australian college of GPs', GPs from the Darwin Urban division of GPs, Australia	Purposeful sample of 8 practices of GPs in the North Tames region, members of the 'Medical Research Council General Practice Research Framework', United Kingdom
**Design**	Quantitative: Questionnaire	Quantitative: Questionnaire	Cross-sectional research: Questionnaire	Qualitative: Focus groups	Qualitative: Semi-structured interviews
**Respondents**	N = 154 GPs Response rate 78%	N = 452 Response rate 67%	N = 294 Response rate 60%	N = 27	N = 24
**Barriers**	**Factors** -Time consuming -Decrease of the art of medicine -Lack of evidence -Experience not taken into account	**Factors** -no skills in critical appraisal -EBM threatens GPs -Time consuming -No access to information -Organisational Chaos -No financial profits -Gaps in evidence -Evidence does not fit general practice -Too much evidence -Evidence hard to implement	**Factors** -Too academic -Decrease of the art of medicine -Movement still young -Gaps in evidence -not applicable to individual patient -Decrease of importance of experience	**Factors** -Reduction of therapeutic freedom -Contradictions in evidence -Not applicable in daily practice -Not applicable to individual patient -Studies too quantitative	**Factors** -lack of time -Lack of information sources -Lack of knowledge and skills -Too much pressure, less motivation -Evidence does not count complexity of situations in practice
	**Actors** Patients -erosion of autonomy	**Actors** Patients: -expectations do not fit EBM -does not except certain advice Colleagues: -Not evidence-based minded Government: -No investments Media: -Counterproductive messages		**Actors** Commercial organisations: -have influence on evidence Patients: -Do not count in terms of risks	**Actors** Patients: -No compliance -Specific cultural background -Specific values and knowledge -Behaviour GP = avoiding conflict -Clientism

**Study Year**	**Scott et al 2000 [6]**	**Freeman et al 2001 [7]**	**Young et al 2001 [8]**	**Ely et al 2002 [9]**	**Putnam et al 2002 [10]**

**Population**	Sample of members from the 'Internal Medicine Society', Australia and New Zealand, participants of an EBM-course program, doctors with a practice in 5 hospitals	Purposeful sample of GPs out of three regions concentrated around a hospital, United Kingdom	1. GPs, participants of a research project on preventive care, selection of those willing to participate, Australia	Sample of GPs in Iowa, United States	Purposeful sample of GPs with a minimum of one year experience, patients with cardiovascular problems, working in the region Nova Scotia, Scotland
**Design**	Quantitative: Questionnaire	Qualitative: 3 focus groups	1. Quantitative: Questionnaire 2. Qualitative: semi-structured interviews	Qualitative: observations	Qualitative: 9 focus groups
**Respondents**	N = 111 Response rate 20%	N = 19	N = 60	N = 25	N = 50
**Barriers**	**Factors** -Lack of time -No access to information -Problems in organisation -Lack of knowledge and skills -GPs not motivated -Not applicable to individual patient -Inconsequence in evidence	**Factors** -Lack of logistic support -Too many habitudes -Decrease of importance of experience	**Factors** -Lack of time -High cost of information sources -Lack of skills -Not applicable in daily practice -Evidence-Based acting = less patients an hour	**Factors** -Lack of knowledge and skills -Too less capacities to implement EBM in practice	**Factors** -Lack of time -Lack of competences -Evidence = dogma, confusing -Not applicable to individual patient -Decrease of importance of experience
		**Actors** Patients: -Does not accept certain advice -Specific characteristics -Asks for certain treatments -Do not always understand evidence-based message Colleagues: -Do not consider the patient in total -Specialist = evidence-based mafia	**Actors** Patients: -Asks for certain treatments -Specific expectations -Do not always understand evidence-based message		**Actors** Patients: -Brings info from internet -Not interested in EBM -Not enough competences to understand EBM -Creates uncertainty in the patient

**Study Year**	**Al-Ansary et al 2002 [11]**	**Shawn et al 2003 [12]**			

**Population**	All GPs out of the region Riyadh, Saudi Arabia	GPs/participants of a national research program on the implementation of EBM,			
**Design**	Quantitative: cross-sectional research, questionnaire	Qualitative: semi-structured interviews			
**Respondents**	N = 559 response rate 86%	N = 15			
**Barriers**	**Factors** -Lack of time -No access to information sources -Limited information sources -No high quality training programs available	**Factors** -Lack of time -Lack of information sources -No access to information sources -Lack of competences -Scientific studies not attractive -Decrease of the art of medicine -Decrease of clinical autonomy -Too much pressure -Inconsequence in evidence -Reliability and generalisation of scientific studies? -Not applicable in general practice -GPs actions based on intuition			
	**Actors** Patients: -Specific attitude	**Actors** Patients: -Values and preferences of patients must be considered Colleagues: -Too less specialists working local Commercial organisations: -evidence sponsored by industry			

**Table 2:** Studies addressing strategies to bridge barriers towards EBM in general practice

**Study Year**	**Hayward et al 1999 [13]**	**Oswald et al 1999 [14]**	**Brassey et al 2001 [15]**	**Markey et al 2001 [16]**	**Alper et al 2001 [17]**	
**Population**	Stratified, randomised sample of GPs in division South of Adelaide, Australia	Theoretical sample of GPs willing to participate, with patients with a non-rheumatic atrial fibrillation, 6 general practices in Cambridge, United Kingdom	GPs who use the information service ATTRACT ('evidence-based summaries to clinical queries'), United Kingdom	All GPs/members of the 'Monash Division of General Practice in the South-East Suburbs' of Melbourne, Australia	2 GPs 2 information-specialists, United States	
**Design**	Action research, Telephonic interviews No control group mentioned	Prospective research design: 6 months follow-up No control group mentioned	Quantitative: Questionnaires, No control group mentioned	Quantitative: RCT	Registration of answers to questions found in medical databases	
**Respondents**	N = 31	N = ?	N = 42 response rate 84%	N = 132 response rate 48%	N = 4	
**Tested interventions**	Set-up of an online support system through which doctors can submit a form with their question(s), being answered by an information specialist	Evaluation of plans of care of patients in patients records, evaluation of current type of care in the framework of criteria of a treatment protocol the doctors made themselves.	Set-up of an online support system through which doctors can submit a form with their question(s), being addressed with a summary of current scientific results	'Academic detailing': introduction in EBM and exploration of knowledge and attitudes by an educative worker in the home practice of the GP	Identification of qualitative databases, being able to answer questions of GPs	
**Conclusion**	GPs found the answers useful to support their clinical decisions. In four of twenty cases the answers had a positive effect on the management of the patient.	Doctors noted their reasons to neglect the recommendations of the protocol very explicit. They pointed at the difficulties of applying the recommendations of the protocol on their individual patient.	GPs appreciate clear summaries of scientific literature. The answers lead to a change in daily clinical practice.	'Academic detailing' leads to a significant improvement in knowledge and understanding of EBM, but does not affect the attitude towards EBM. It is not clear whether academic detailing can motivate practitioners to change their clinical practice.	Existing databases are capable of answering most questions of practitioners. However, a lot of gaps in scientific knowledge should still be addressed.	

**Study Year**	**Del Mar et al 2001 [18]**	**Swinglehurst et al 2001 [19]**	**Fritsche et al 2002 [20]**	**Greenhalgh et al 2002 [21]**	**Al-Ansary et al 2002 [11]**	**Schwartz et al 2003 [22]**

**Population**	GPs with an education in information programs, Australia	GPs of the region Fulham and Hammersmith, United Kingdom	Participants of a course program on EBM, Berlin, Germany	Selection of doctors in the field of primary care	All GPs out of the region Riyadh, Saudi Arabia	3 GPs from one practice, coaching junior doctors for the university of Detroit, United States
**Design**	Action Research, Questionnaire No control group mentioned	Descriptive pilot study: Questionnaire and semi-structured interviews, No control group mentioned	Quantitative: pre-post design	Case studies combined with qualitative research methods	Quantitative: cross-sectional research with questionnaire	Prospective research design: Registration of search results, 3 months follow-up
**Respondents**	N = 71	N = 34 response rate 34%	Two Cohorts N 1999 = 82 N 2000 = 50 N 2001 = 71	N = ?	N = 559 response rate 86%	N = 3
**Tested or interventions**	Set-up of two information desks to assist practitioners in their search for medical literature (Quest and Aqua)	Set-up of a clinical information system (helpdesk) to support practitioners in taking their clinical decisions	Intensive 3-day course in EBM	Comparison of an academic feedback system for practitioners and a practice-oriented feedback system	*Suggested intervention: *Training programs in searching scientific literature and critical appraisal, the use of clinical guidelines and protocols	Searching for evidence during the encounter with the patient
**Conclusion**	An information desk is useful to assist practitioners with their search. However, a cost-utility analysis should be undertaken to evaluate both information desks.	The helpdesks succeeds in creating a better access to 'evidence' for practitioners. GPs are satisfied with the system, but the number of users is very low. For those who used it, it actually led to a change in their clinical practice.	The course led to a significant improvement of knowledge and skills towards EBM.	A good information system simultaneously provides a search engine for researchers and a search engine for practitioners.	Concrete actions to implement EBM in the field of health care are very necessary.	Time that must be invested in a search for answers is an important barrier to use information systems during patient encounters. It can be bridged by high quality summaries of literature. Faster internet connections are necessary.

## Methods

An inductive qualitative study was conducted (September 2002 – June 2003) using four focus groups with a total of 31 GPs. Purposeful sampling was used to recruit participants. Two major criteria were used to select the participants: (1) variability in interest towards EBM and (2) variability in expertise with respect to EBM. One group of 7 academics was chosen because of their status of being good informants on EBM. Group 2 consisted of 7 GPs recruited from different local peer groups in a major Flemish city. Group 3 consisted of 6 GPs recruited from an interuniversity postgraduate course in EBM. Group 4 consisted of 11 GPs from a local peer group in a small town.

**Figure 1:**
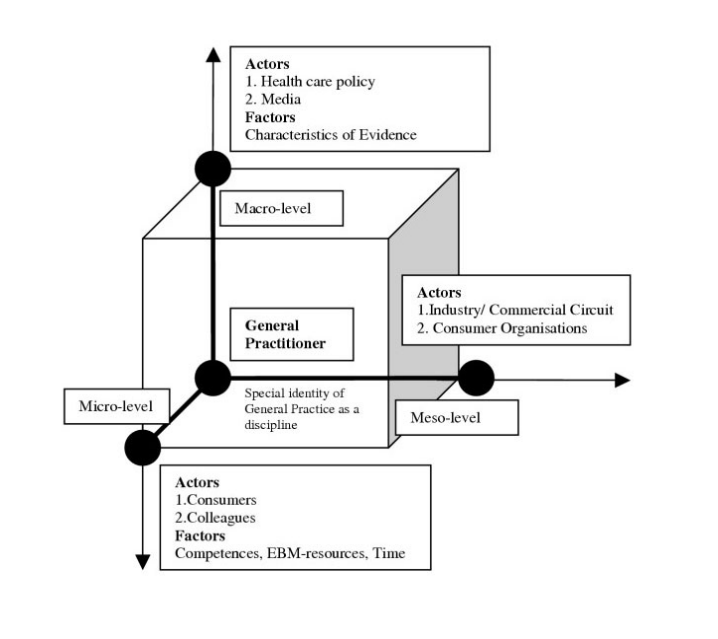
Classification scheme of influencing 'Actors' and 'Factors' on different levels.

A professional moderator was hired to facilitate the focus group discussion using a semi-structured interview guide. One researcher took notes on non-verbal actions of participants. Four general topics were discussed: 1. Knowledge and understanding of EBM; 2. Applicability of EBM in general practice; 3. Specific barriers to implement EBM; 4. Suggestions for bridging the barriers. At the end of the focus group (1.5–2 hours) GPs were asked to complete a short questionnaire collecting demographic data (table 3). Each focus group was recorded and transcribed verbatim. Two independent researchers identified the important concepts by coding the first two transcripts separately. No major interrater inconsistencies were found. Data collection and analysis were guided by a combination of a 'between-case' analysis [23] and an inductive strategy based on 'grounded theory' approach [24]. In the 'between-case' analysis we classified the content of all focus groups (1 group = 1 case) in a matrix, looking for themes that cut across the different cases. Concepts were classified in different levels: a micro-, a meso- and a macro level (table 4). To check the consistency of the matrix all individual ideas were then attached to a board and categorised by a group of researchers from three different disciplines (medicine, sociology, andragology). No theoretical framework was proposed to the researchers in advance. Starting from the original data, barriers and strategies were then labelled as clusters of influencing 'actors' and 'factors' (table 4) towards the ability of GPs to implement EBM. To gain a certain level of abstraction in the empirical material insights of both analyses were used to develop a classification framework (figure 1), which will be further explained in the results section.

**Table 3:** Demographic data of GP participants (n = 31)

		**()**
Sex: Male (%)	22	71%
Female (%)	9	29%
Average Age (Min/Max)	45.9	25/67
Average year of graduation (sd)	1982	9.9
Province: Antwerp(%)	11	35.5%
Brabant (%)	15	51.6%
Limburg (%)	1	3.2%
Brussels (%)	4	10.7%
Practice: individual (%)	11	35.5%
group (%)	20	64.5%
Average years of practice (sd)	18	10
% present in practice: full-time (%)	26	83.9%
part-time (%)	5	16.1%
Affiliated with university/scientific organisation: Yes (%)	18	58.1%
No (%)	13	41.9%

**Table 4:** Explanation of the concepts used to build the classification scheme

**Micro-level: **problems and interventions related to the individual practice of GPs, with a direct impact on the personal acting of the GP in the consultation setting.
**Meso-level: **problems and interventions related to organisations and institutes, for example scientific institutes, consumer organisations, enterprises, universities, small-scale formal and informal communication circuits etc.
**Macro-level: **problems and interventions with an impact on the broader social environment or related to a policy level, for example government, the media etc.
**Actors: **all acting persons or entities in the field of health care that influence the ability of GPs to think and act according to the principles of EBM. The main characteristic of an actor is 'interaction' with the GP. Interaction can be described as a process that occurs when two or more actors interchange and have an effect upon each other.
**Factors: **all elements that influence the ability of general practitioners to think and act according to the principles of EBM and to which interchange and interaction cannot be attributed as a characteristic.

## Results

The classification scheme focuses on influencing 'actors' and 'factors' at different levels in health care that might have an impact on the ability of GPs to think and act according to the principles of EBM (figure 1). All EBM-related barriers and strategies, mentioned by the GPs, were classified as an 'actor' or a 'factor' on a micro-, meso- or macro-level. On the micro level (individual practice) we identified two 'actors' (patients and colleagues) and three 'factors' (competences, time and EBM-resources) that have an impact on the intention of GPs to handle clinical problems according to the principles of EBM. On the meso level (institutional level) two 'actors' were identified to affect implementation of EBM: commercial organisations and consumer organisations. Health care policy and the media were the influencing 'actors' on the macro level (broader social and political environment). The 'factor' 'characteristics of evidence' was also categorised on the macro level, because of its relevance to the international scientific community.

### 1. Problems and interventions related to the special identity of general practice as a discipline (table 5)

**Table 5:** Statements related to the specific identity of general practice as a discipline

Reference number*	**Statement**
1:23.1*	(EBM) is a new phenomenon... the fundamental question we have to ask ourselves is how does that part of reality, that scientific approach of health and disease fit in the totality of the GP as a person...I do have the strong impression that we are acting too fast, without taking time to reflect on our actions...
4:23.3	For me the most difficult thing is getting the diagnosis right and evidence-based. Cough, ... okay cough, but cough is a very complex item... you can't look at it with an evidence-based eye alone. I think that clinical aspects are very important indeed...
1:153.4	... Maybe that is a task for universities to make a serious scientific-philosophic analysis of what is called EBM. A strength-weakness analysis, making the borders clear so that we can resist critics...and becoming dissidents of our own convictions.
4:36.1	I think everyone builds some decision trees based on existing knowledge and experience... EBM is another one. We should take the step to try it out at least. But it won't be easy to change a habit in no time.
	*The number of the focus group (first number) does refer to the place of the interview within the hermeneutic unit of the software programme ATLAS-TI, hence it is likely that this number exceeds the number of focus groups reported due to test groups or try-out files that are used within the same hermeneutic unit. *first nr. = focus group/second nr. = citation/third nr. = respondent

In the focus group interviews one of the important issues was: does the EBM-paradigm fit our specific way of acting and the problems that occur in our daily practice (1:23.1 – statement 1 in table 5)? Many GPs stated that their acting is built on intuition, habits and previous experience. They also indicate hat it is often impossible to handle the care for patients completely evidence-based, because the patients' symptoms are often vague and GPs are confronted with a broad spectrum of different questions and diseases (4:23.3). A continuous update on scientific information is not feasible. According to several GPs a possible strategy for improving the use of evidence will need a better demarcation of the GPs tasks and responsibilities. The rather broad spectrum of health care problems they are confronted with makes it hard to keep up to date with current knowledge. Furthermore they stated that their intellectual position must be strengthened (1:153.4). Many GPs think it is worthwhile to be stimulated and encouraged to reflect on individual decisions, patterns of prescribing and perceptions of therapeutic freedom (4:36.1). Furthermore, GPs consider very important the impact of 'evidence-based thinking and acting' on outcomes (improvement of mortality and morbidity rates, better quality of life,...) for patients to be assessed in order to keep them motivated to implement it in practice. Despite the fact that many patient outcomes can not be easily fit in existing research designs on the effectiveness of clinical practice, the participating GPs do feel the need to be aware of these outcomes in order to maintain the efforts needed for EBM.

### 2. Problems and interventions at the micro-level (table 6)

**Table 6:** Statements related to the micro-, meso- or macro-level

**Reference Number**	**Statement**
**MICRO **4:134.2*	Look, it is like you just said: if people come to me and say:" I want that blood analyses!", I will do it...
6:30.2	Information that is brought in by patients from the internet is not evidence-based in most of the cases. It conflicts with what we know and are willing to provide. It is a tough discussion... (A:8,9)*
1:53.2	...People who take statines for several years for example... at one point in time you have to say:" quit using them because the reason I prescribed it for you, three years ago, it does not have any value today (B: laughing). I know you took it three years, but it is okay, quit using them immediately..."
4:148.5	But take the discussion about anti-hypertensions for instance. I expect from a specialist that he knows what he is talking about, it is his job. But sometimes they are just promoting medical products, they advertise... and I think he will have had his pleasant trip organised by a company... that feeling is hard to deal with. I do not feel like they are acting evidence-based.
1:65.6	The last month I got two patients back... look, your patient does not fulfil our criteria and so he does not have to come. And I think: "Is that the kind of medicine I will be forced to do? That person comes with his complaints, whether he is fulfilling my criteria or not. But that will be the future task of the GP: helping the people who do not fit the criteria of the specialists.
1:56.1	Sometimes I have the feeling that those people who are not connected to a university or an academic hospital ... the ones that are more modal... when they take the word, when they take over, it is easier. Late adapters get convinced and start moving.
4:180.3	A GP who works alone needs a contact to people who can guide him in a certain way, because there is to less time to figure it out yourself. That step can be made very easily, because you know who can be contacted and so on...
4:181.2	Some time ago one was talking about independent educators from government for outreach visits. Now that would be interesting, for instance to visit each GP for half an hour – once a year – like commercial representatives. They can explain where the good sites are, how they are used, show all options. And then after half a year they can come back to see how it went, did you use it? And now let's see how you can use it during your consultations...That would be interesting...
1:126.4	If one would like to know something about a certain topic, one interviews professor bla bla bla. He will know what it is all about... instead of organising a social debate, in which complexity of EBM can be explained to the public. Government can play an important role in that.
	
**MESO **6:99.9	There's too much fragmentation: evidence-based journals, scientific institutions, organisations for EBM...all trying to promote evidence-based acting. It would be a good thing for those initiatives to melt together.
1:152.5	The booming business in the US... right now they are setting up commercial structures to make products of other companies evidence-based. That's business... not developing drugs, but developing evidence and set up large studies... sell them as evidence, to impress the rest of the world.
5:73.3	I held an archive of all medical information for one month for a talk a prepared for a governmental organisation. I had such a big amount of information! It is incredible how much we are influenced by commercial institutions and it is in no way comparable with the scarce information we get from independent sources. I think government must take a more active role in providing that kind of information.
1:101.4	There is a culture rising where patients are defined as consumers in a health care system. But often messages of consumer organisations are counterproductive, because they are not methodologically sound.
	
**MACRO **1:188.6	I had a patient in my office lately that went to a specialist who said:" I have to talk to you for five more minutes because I need to gain an average of 10 minutes for a consultation (B: laughing)*. A rule from management.
4:102.2	The system is counterproductive for EBM. Some gynaecologists and GPs make a cervical smear every half year. If I tell that in the Netherlands they will have a good laugh, because they only do it once in three years. In Flanders women become 'smeared' far to often, just because it is easy money.
4:132.6	And I think government may be firm about that. If they say one cervical smear each three years, it means that there is only one pay-back to the patient. If the doctor talks you into more than one, ... sorry, you have to pay for it yourself (I:7,5)
1:212.5	It is the good care for the patient that should guide judgements about clinical practice and should be the most important parameter,... the degree of practicing evidence-based medicine can not be the sole norm (A:all)
1:98.2	... if you hear things like allowing drug commercials on television. Well, that's like cleaning the floor while someone is painting the ceiling, because they heard on the commercials how good this drug is... and you have to explain, based on evidence, that it is not... and tell your story over and over because they all have seen it on television.
6:27.8	Yes, but all is presented so over-simplified...it makes consultations more difficult. In the past we were God himself and said: here take clamoxyle and go home. Our scheme was simple back then. On this side science is sitting and on the other side the dependent patient.
1:149.3	Economic thinking would be using the means we have as efficient as possible, based on transparent choices. We are not there yet and that's a reason why doctors should sit around the policy table too, to negotiate. We have to prevent letting public servants and insurance companies take decisions about health care on their own, because that indeed would be dangerous.
5:56.3	..."The publishers feel that it will be helpful for clinicians to know whether their uncertainty sustains from the gap in the evidence rather than the gap in their own knowledge." So for the most questions there will be no clear answer, not because you do not know it but because evidence simply does not exist. And than it is up to you to take decisions.
6:11.8	I often ask myself... that EBM process is so slow-moving. By the time everyone has picked up the new evidence there probably will be a second movement that will reject those findings or will look at them from a different point of view.
	*first nr. = focus group/second nr. = citation/third nr. = respondent – A = agreement followed by respondent – B = behaviour (software programme ATLAS-ti)

#### Actor: patients

The interaction process with patients has, according to many participants, an impact on the possibility to implement EBM in practice. Many patients have clear expectations on how they wish to be treated. This forces GPs in a position adapting to the patients' preferences, rather than to behave in an evidence-based manner (4:134.2). GPs state that the emancipation process of patients has blurred the autocratic position of doctors and demands for different behavioural strategies. Despite the fact that doctors consider their patients as well-informed, they lack the knowledge of EBM (6:30.2). Many GPs refer to the difficulties of patients to understand the rapid changes and insights in medicine (1:53.2). To dissolve this problem, most of the GPs are willing to take responsibility to create a safe environment in which the evidence-based story can be taught to the patient, for example by searching for evidence together through an evidence-based internet approach during consultation. Consumers need education, in which they can be taught to behave critically towards medication and media. According to several GPs consumer organisations could play an important role here.

#### Actor: colleagues

Conflicts with colleagues seem to be common. GPs clearly express the opinion that specialists' thinking is too commercial (4:148.5). This implies they are promoting and using products based on a commercial rather than an evidence-based approach. There is a general feeling that specialists who do act evidence-based should be protected. GPs also feel strongly for building networks with reliable colleagues. The fact that patients still consider the advice of a specialist more valuable is also brought forward. GPs are very much concerned about the fact that their role will become reduced to treating those patients who do not fit the criteria of specialists (1:65.6). Furthermore, in educational programmes more lectures and workshops should be given by colleagues who are not explicitly connected to universities, making EBM more of a real-life business (1:56.1).

#### Factors: time, EBM-resources and competences

Lack of time, EBM-resources, knowledge and skills are the barriers that are most recognised in existing literature. They recur during the focus group interviews. Many GPs think it is worthwhile to create a helpdesk system or a central on-line information system that provides answers to specific questions (4:180.3). On-line sources should be easy to navigate and free of charge. Guidelines and Critical Appraised Topics would be a great help. A select team of GPs should be paid to develop them. Educational programs should focus on an integration of EBM-principles in medical school and further training courses. Specialised EBM-teams should be established. The idea of outreach visits by independent educators, paid by government, gains approval by many GPs (4:181.2). As a lot of existing EBM material does not reach the GPs, they suggest to put more efforts into getting this material under their attention. Furthermore, it will be important to change GPs attitudes towards EBM. Many GPs state that EBM remains too theoretical and authority based (1:126.4). It restricts clinical practice, limiting the choices. Resistance should be broken down, for instance by finding inspiration in change management techniques, as indicated by some participants.

### 3. Problems and interventions at the meso-level (table 6)

The EBM movement is relatively new in Flanders. At this stage different organisations undertook efforts to stimulate EBM, for example universities, the Belgian Centre for Evidence-Based Medicine and other occupational groups. Some GPs criticise the fragmentation of initiative, which does lead to an organisational chaos in the field. GPs do see a role for government in providing a network which tightens all different institutions together (6:99.9).

#### Actor: commercial organisations

Several GPs criticise the fact that educational programmes and the development of guidelines is sponsored by commercial organisations, for example pharmaceutical firms and their representatives (1:152.5). Questions do rise about the honesty of their message, in which EBM is used as a fashion term. Moreover, GPs receive a lot of information from different sources. Only a small part of the information is independent and unfortunately not easily identified in the information overload (5:73.3). Suggestions are made to label independent information, distinguishing it from less relevant information. Conflicts of interest must be clearly stated. Many participants feel that spreading a uniform EBM message by all actors in the field of health care is very important and that this specific approach should also become a necessity for the commercial organisations.

#### Actor: consumer organisations

GPs in the sample worry about the quality of the messages spread by consumer organisations, for example self help groups. In order to stimulate EB-practice more attention should be paid to the content and form of the messages and the methods used (1:101.4). According to several GPs consumer organisations could play a major role in distributing evidence-based messages to patients that are screened for quality and in measuring their satisfaction with evidence-based treatments.

### 4. Problems and interventions at the macro-level (table 6)

#### Actor: health care policy

Belgium has a particular way of financing the GPs. GPs are most of the time independent entrepreneurs, who are financed by the number of medical acts defined in the nomenclature. Several GPs raise their voice about 'the policy system' that works counterproductive for an evidence-based approach. The time spent on research and information seeking behaviour is not paid for. One focuses necessarily on the amount of consultations, rather then evidence-based acting, and doctors adapt to the protocols they are forced to follow (1:188.6). Consultations lead to a higher income for a doctor but, as indicated by several GPs, often also to unnecessary practices (4:102.2). According to some participants a fixed income could be a solution, as well as financial incentives for extra time that is invested in acting evidence-based. Reimbursement systems should be consequent with what is promoted regarding to EBM (4:132.6). Some GPs criticise the fact that EBM seems to develop as a reductionist model to judge results of clinical acting. Most GPs think that broader outcome-measures should be taken into account, for example quality of life (1:212.5). Some concerns are expressed about the coaching of GPs to implement evidence. Too easily, government assumes that all doctors are comfortable with the evidence-based paradigm. GPs are given the noble task of being the messengers, without having enough structured information and without being enough knowledgeable for that 'new' role. Therefore many voices are raised to improve the distribution of evidence-based information.

#### Actor: the media

The mass media are identified as a counterproductive force, because products or services are being promoted that are not always supported by evidence (1:98.2). Patients seek answers to their questions on the internet. The quality of this information is doubtable. This changing information seeking behaviour has an impact on the relation between GPs and patients. Some GPs state that patients become negotiating partners (6:27.8). Starting a social debate could be a first step to promote coherence between different parties of interest involved in the production, interpretation, use, dissemination and promotion of evidence. In defining a rational policy towards EBM GPs find it important to be involved in the process and to integrate late-adapters in the evidence-based movement (1:149.3). The media could play an important role in representing their voice in the economic choices made to support an evidence-based health care system, since these choices are often made by governmental institutions only.

#### Factor: sub-optimality of 'evidence'

The sub-optimality of the evidence provided is frequently stated as a barrier to the implementation of EBM. Contradictions between similar evidence-based studies are common. Some of the studies that are promoted as 'evidence-based' are not up-to-date. Several GPs point towards the lack of evidence for many of their problems (5:56.3). There is also a time-delay between the research process and the actual adaptation of the new insights in the field (6:11.8). No explicit suggestions to overcome these barriers are given by the participants.

## Discussion

The results presented in previous sections give an overview of issues mentioned by different GPs during the focus group sessions. Compared to what already was known from previous qualitative research (table 1.2) this study adds two more 'actors' that influence the implementation of EBM in general practice: consumer organisations at the meso-level and health care policy at the macro level. Providing logistic and educational support seems to be important to participants in previous research programmes, as well as for Flemish practitioners. Recent reviews on the effects of decision support systems to support evidence-based practice conclude that these strategies improve practitioners' performance [25,26]. Practitioners start using online evidence during consultations much more than previously reported [27,28]. Educational programmes have also proven to impact on practitioners' competences [29,30]. However, they should be moved from the classroom to clinical practice [31]. The approach for implementing EBM in general practice needs to start from the specific characteristics of the profession. Despite the fact that GPs are keen to consider evidence in clinical decision making, there is a widespread belief that intuition plays a vital role in primary care [32]. GPs are not likely to change this belief until the impact of the evidence-based thinking and acting on patients' outcomes is assessed.

Apart from these insights the focus group research reveals that interventions should also focus on communication, rational policy making and the building of networks between different 'actors' to impact on an actual implementation of EBM in practice. The analytic classification proposed in this paper has the advantage to identify the complexity of problems and by consequence to identify and focus on interventions. To our knowledge no other study in the field of difficulties in the implementation of EBM in general practice has proposed a theoretical framework wherein barriers can easily be located and which naturally leads to the development of strategies to tackle them at the right level. We feel the framework is of relevance to the international community of GPs and those who promote EBM in this context. The classification model as presented seems to be a useful tool to orient change management processes.

Some specific barriers for GPs are directly linked to the specific characteristics of the Belgian health care system. These characteristics should be taken into account in other countries or regions. Considering this aspect we want to warn for generalisations even in Belgium. Although the organisation of the health care system is a federal issue, the results presented could be biased since health care traditions are culturally diverging in Flanders, the Walloon provinces and Brussels. However, these contextual issues are fundamental to address when developing policies or interventions.

The issue of generalisation brings us to some other methodological considerations. The 31 general practitioners who participated in this study formed a small sample of Flemish GPs. The sample was neither random nor representative. Given the basis on which most of the GPs joined the study we assume that their interest in EBM would be greater than that of their colleagues in the field. However, we are convinced that precisely this positive attitude and experience revealed knowledgeable results to contemplate further about interventions facilitating an evidence-based approach. The focus group methodology was considered appropriate to identify barriers and suggestions to overcome them. Methodological rigour was assured by using a combined approach of 'between-case analyses' and an inductive strategy based on 'grounded theory approach'. The first two focus groups were coded by two independent researchers and the analysis was checked and discussed with two senior researchers. However, when assessing the outcomes of the focus groups it was noticed that saturation was not reached. The last focus group interview still generated seven new ideas: five new barriers and two more interventions. Therefore we cannot guarantee that all factors, actors and suggestions were identified. More methodological research on predictive models for saturation points in qualitative research is necessary.

Another interesting methodological issue is that we found a high level of 'agreement' between participants. A possible explanation for the lack of 'extreme cases' could be that the topic under study (the implementation of EBM) was relatively new to many participants. Conversation in the focus groups were about 'recognition' and 'explanation' rather than 'debate' and 'opposing opinions' about suggestions. This probably explains why we inventoried more barriers than suggestions on interventions and strategies.

## Conclusion

The classification model can be used as a frame of reference to initiate change management and policy interventions. For a successful implementation of EBM different 'actors' should be mobilised at different levels, as they are able to prevent GPs from acting evidence-based. However, potential conflicts between 'actors' can be bridged by (1) creating the necessary communication platforms between actors at different levels and within different disciplines (including patients, policy makers, researchers, consumer organisations, representatives from the media, commercial organisations, primary care practitioners, secondary care practitioners etc.), (2) communicating a consistent and uniform message on the principles of EBM and (3) modifying the current health care system towards a more evidence-based approach (for example an update of the reimbursement system, incentives that stimulate evidence-based acting, (re)opening the discussion on a fixed income vs. an income based on consultations). Future research projects should focus on the changing relations between GPs and other 'actors'. Furthermore, (4) qualitative educational programmes should be offered, ideally within working hours, with a reasonable compensation for the income lost and organised by independent organisations, (5) access to EBM-resources should be free of charge, easy accessible and whenever possible provided in the mother tongue of the practitioners and (6) gaps in knowledge need to be addressed by researchers world wide. A better understanding of the complex relationships as proposed in the conceptual framework (figure 1) and the 'factors' influencing the implementation of EBM will hopefully lead to the funding of research projects in which a set of strategic priorities can be inventoried and worked out to support and stimulate a coherent evidence-based approach within the health care system. In this important negotiation process all identified actors in the field of health care have to be involved.

## Competing interests

The author(s) declare that they have no financial competing interests. However, answers to questions could have been influenced by a convenience bias, as the participating GPs were aware that the focus groups were carried out by a research team known to be in favour of EBM.

## Authors' contributions

KH set up the focus groups, coded the manuscripts, analyzed the qualitative data and drafted the manuscript. BA coordinated the research process and participated in the design of the study. EV assisted in the coding process and participated in the data-analysis. ML helped developing the conceptual framework. AD and FB interpreted the results and commented on the draft version of the manuscript. All authors read and approved the final manuscript.

## Pre-publication history

The pre-publication history for this paper can be accessed here:


